# Association between life’s essential 8 and periodontitis: a population-based study

**DOI:** 10.1186/s12903-023-03816-z

**Published:** 2024-01-04

**Authors:** XingJin Chen, JiangLing Sun, ChongWen Zeng, FuQian Jin, Shu Ma, Jukun Song, Zhu Chen

**Affiliations:** 1 School of Stomatology, Zunyi Medical University, Zunyi, China; 2 Department of Endodontics, Guiyang Stomatological Hospital, 253 Jiefang Road, Nanming District, Guiyang, 550005 China; 3 Department of Oral and Maxillofacial Surgery, The Affiliated Stomatological Hospital of Guizhou Medical University, No. 9, Beijing Road, Yunyan District, Guiyang, 550002 China

**Keywords:** Life’s essential 8, Periodontitis, NHANES, Cross-sectional study

## Abstract

**Background:**

The American Heart Association has developed a novel cardiovascular health indicator called Life’s Essential 8 (LE8). However, no one has reported using LE8 to assess periodontitis. This study aimed to investigate the association between LE8 and periodontitis in American adults.

**Methods:**

Data from the National Health and Nutrition Examination Survey (NHANES) from 2009 to 2014 were used for this investigation. LE8 was the independent variable, and it is divided into three grades: high, moderate, and low. Periodontitis was the dependent variable, and the classification of periodontitis was based on the criteria of Eke in 2012. Multivariable logistic regression models were used to explore the relationship between LE8 and periodontitis.

**Results:**

A total of 9,039 participants with an average age of 52.16 ± 14.21 years were enrolled in this study, of whom 48.29% were male and 51.71% were female. The mean and standard deviation of LE8 was 66.29 ± 14.57, and the prevalence of periodontitis was 50.48% overall. The LE8 score and periodontitis in the fully adjusted logistic regression model showed a negative correlation (OR = 0.98; 95% CI, 0.98–0.99, *p* < 0.001). This result persisted when Life’s Essential 8 was categorized into low, moderate, and high groups. Compared with those in the lowest group, those in the highest LE8 group had a 47% decreased risk of periodontitis (OR = 0.53; 95% CI, 0.46–0.66, *P* < 0.001).

**Conclusions:**

This cross-sectional investigation revealed a negative relationship between the LE8 score and the likelihood of periodontitis.

**Supplementary Information:**

The online version contains supplementary material available at 10.1186/s12903-023-03816-z.

## Background

Periodontal disease, which includes gingivitis and periodontitis, is highly prevalent in adults [1]. Gingivitis is considered an early form of periodontal disease. Further aggravation of this condition, causing loss of attachment, results in periodontitis [2]. Periodontitis is a chronic inflammatory disease that causes the destruction of periodontal supporting tissues. Due to the presence of long-term inflammation, it can spread deeper into the periodontium and alveolar bone, causing loss of the alveolar bone, which can lead to loosening and loss of teeth [3–5]. It is reported that more than 40% of individuals in the United States suffer from periodontitis [6]. In addition, according to China’s fourth national oral health survey, the prevalence of periodontitis among participants aged 55–64 is as high as 69.3% [7]. Moreover, a host of systemic disorders, such as hypertension, pneumonia, diabetes mellitus, cardiovascular disease, and adverse pregnancy outcomes, are linked to periodontitis [8–11]. Obviously, periodontitis represents a significant public health burden.

In 2010, the American Heart Association developed a strategy to promote population and individual health. It centered around creating a novel and actionable definition of cardiovascular health (CVH). The initial indicator used to assess cardiovascular health was called Life’s Essential 7 (LE7), which was later updated to Life’s Essentials 8 (LE8) in July 2022 [12]. Compared to LE7, LE8 adds a sleep metric. It was updated to include diet, nicotine exposure, lipids, and blood glucose [13]. Domestic and international studies have shown that LE8 and cardiovascular health are highly correlated. Higher LE8 scores are associated with lower cardiovascular disease [14–18]. Originally used as an indicator to assess cardiovascular health, some scholars have found that LE8 can also be used as an indicator of other diseases. For instance, Chen H, Tang H, et al. [19] found that adherence to higher LE8 levels in individuals with CKD (chronic kidney disease) is associated with a reduced risk of all-cause and cause-specific mortality. Wang L, Yi J, et al. [20] found that higher LE8 scores are associated with lower odds of nonalcoholic fatty liver disease (NAFLD) among adults in the United States.

It has been reported that oral health is closely related to general health [21, 22], including cardiovascular health [23, 24]. In previous studies, LE8 was primarily used to assess cardiovascular health, and a strong link was found between them. However, no one has used LE8 to evaluate periodontitis. Therefore, in this study, we explored the relationship between LE8 and periodontitis to bridge this information gap.

## Methods

### Data source

The National Health and Nutrition Examination Survey (NHANES) 2009–2014, a publicly accessible database in the United States, provided the data for this study. The NHANES database contains information on demographics, diet, exams, lab tests, questionnaires, and data with limited access. Limited access data requires the submission of an application before it can be used. The National Center for Health Statistics Research Ethics Review Board approved the study’s methodology because all participants provided written permission at the beginning of recruitment [25].

### Study participants

**Fig. 1:**
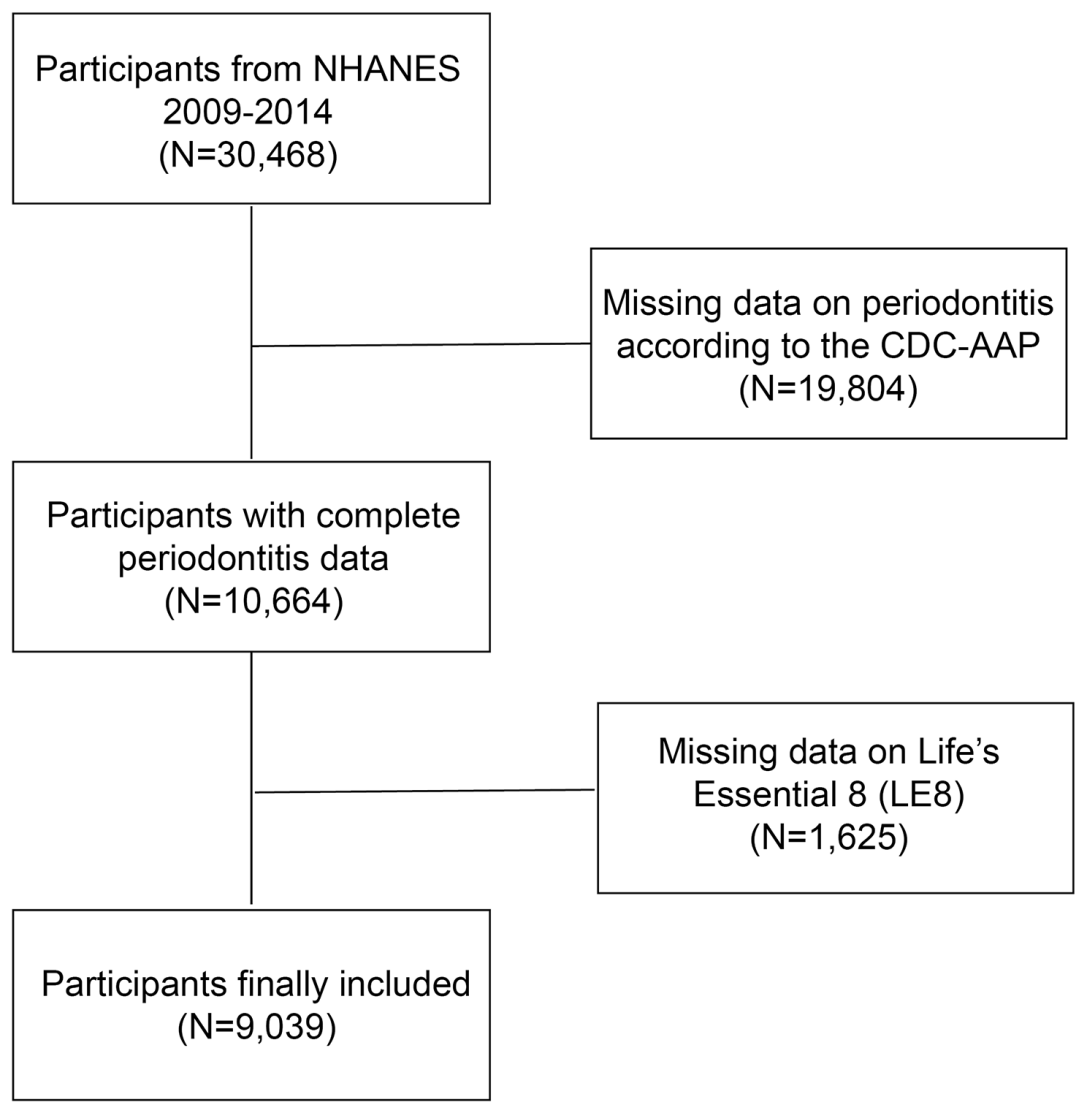
Flow chart of participant selection

According to the NHANES guidelines, only subjects 30 years of age and older received periodontal examinations [26]. Therefore, in this research, exclusion criteria included (1) individuals who were less than 30 years old and missing all teeth and (2) incomplete LE8 scores (the absence of any of the four health behaviors or four health factors). A total of 30,468 participants from NHANES 2009–2014 were selected. Based on this, we removed 19,804 participants without complete periodontitis data who did not receive a full periodontal examination, and 1,625 participants without relevant LE8 data. Finally, 9,039 participants took part in the study in total (Fig. 1).

### Definition of Life’s essential 8 (LE8)

The American Heart Association defined a novel construct of cardiovascular health to promote and maintain human health: Life Essential 8 (LE8). Four health behaviors (diet, physical activity, nicotine use, and sleep quality) and four health factors (body mass index, blood lipids, blood glucose, and blood pressure) are included in LE8. A new scoring methodology with a range of 0 to 100 points is used for each indication [12]. In this study, participants with the LE8 score of 80–100 were considered to have high LE8 scores; those with a score of 50–79 were considered to have moderate LE8 scores; and those with a score of 0–49 were considered to have low LE8 scores. Diet indicators were evaluated by the Healthy Eating Index (HEI) 2015; questionnaire data collected physical activity, nicotine exposure, sleeping situation, and diabetes situation; blood lipid and blood glucose information were available in the laboratory data; and blood pressure, height, and weights were measured on the mobile examination center (MEC). The BMI was determined by dividing the weight in kilograms by the square of the height in meters.

### Definition of periodontitis

**Fig. 2:**
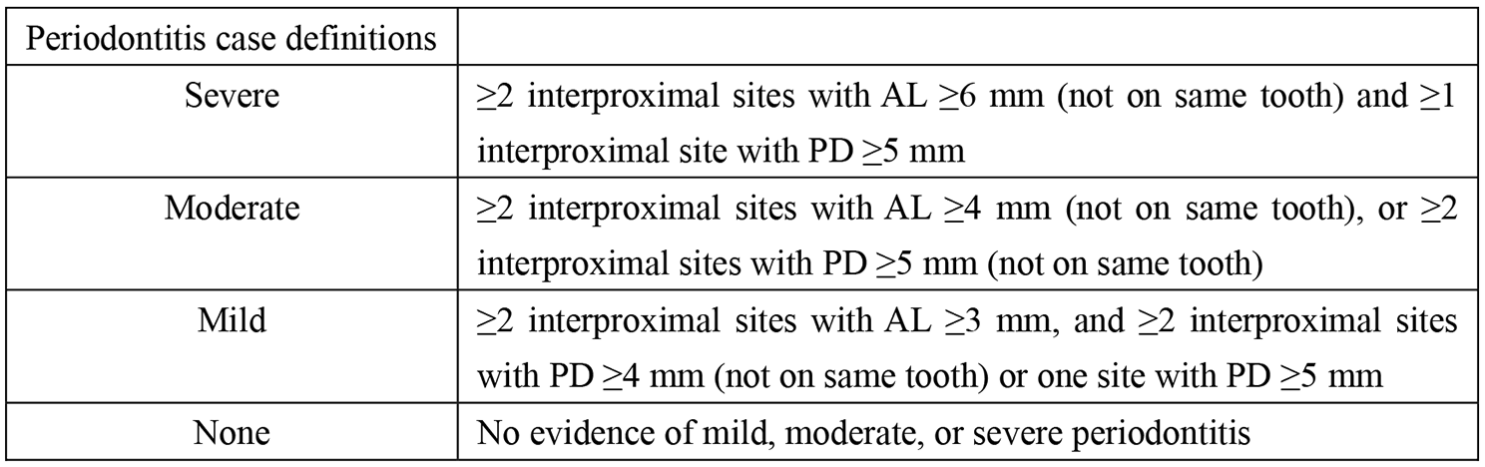
CDC-AAP periodontitis case definitions

In this study, periodontitis was a dependent variable. To ensure data quality, the health technologists who were assigned to conduct the periodontal examinations were intensively trained before the survey began. The main tool used to perform a periodontal examination is the periodontal probe, which is marked with different scales that allow direct observation of the measurements and facilitate the evaluation of the diagnosis [27]. A full-mouth periodontal examination was performed on participants over 30 who had at least 1 natural tooth [26]. Based on the conclusions of Eke, a grade for periodontitis severity has been provided according to CDC-AAP (the Centers for Disease Control and Prevention and the American Academy of Periodontology): severe, moderate, mild, and none (Fig. 2). Severe periodontitis was defined as ≥ 2 interproximal sites with AL ( attached level) ≥ 6 mm (not on the same tooth) and ≥ 1 interproximal site with PD (probing depth) ≥ 5 mm; moderate periodontitis was defined as ≥ 2 interproximal sites with AL ≥ 4 mm (not on the same tooth), or ≥ 2 interproximal sites with PD ≥ 5 mm (not on same tooth); mild periodontitis was defined as ≥ 2 interproximal sites with AL ≥ 3 mm, and ≥ 2 interproximal sites with PD ≥ 4 mm (not on same tooth) or one site with PD ≥ 5 mm [28]. In this study, we grouped mild, moderate, and severe periodontitis into one category (having periodontitis), and no periodontitis in another.

### Covariates

This study also took into account the covariates of sex, age, race, annual family income, education level, marital status, alcohol consumption, floss use, depression, arthritis, asthma, cardiovascular disease, and gout, which may have an impact on the relationship between Life’ Essential 8 and periodontitis [29–31]. Age was a continuous variable, ranging from 30 to 80 years. Race was divided into five categories: Mexican American, other Hispanic, non-Hispanic White, non-Hispanic Black, and others. Annual family income level was classified as low (below $25,000), moderate ($25,000 to $75,000), and high (over $75,000). Educational levels were categorized into two groups: high school and below, and above high school. Marital status was classified as married, widowed, divorced, and others. Alcohol consumption was defined by the National Institute on Alcohol Abuse and Alcoholism (NIAAA) as none, moderate (1 drink per day for women and 1–2 drinks per day for men), heavy (2–3 drinks per day for women and 3–4 drinks per day for men), and binge (4 drinks per day for women and 5 drinks per day for men) [32]. Floss use was divided into 4 groups: never for 0 per week, rarely for 1–2 days per week, moderately for 3–5 days per week, and frequently for 6–7 days per week [33]. Depression was measured using the Patient Health Questionnaire-9 (PHQ-9). In primary care and other medical settings, the PHQ-9 is a nine-item questionnaire used to test for depression. The first study on the PHQ-9 set the conventional cutoff score as 10 or above for screening to identify potential depression [34]. Self-report questionnaires collected arthritis, asthma, cardiovascular disease, and gout information [35]. A positive response to any of the following statements was considered to be indicative of cardiovascular disease: “ever told you had congestive heart failure,” “ever told you had coronary heart disease,” “ever told you had angina/angina pectoris,” “ever told you had a heart attack,” or “ever told you had a stroke” [36].

### Statistical analysis

We utilized EmpowerStats (version 2.0) and the statistical software package R (version 4.1.3) to merge data (The primary data are placed in Supplementary Material 1) and used Adobe Illustrator (version 2021) to create the images in this paper. Categorical variables are presented as percentages and continuous variables are presented as standard deviations. Chi-square tests and Kruskal-Wallis tests were used to determine whether there was a significant difference between different LE8 groups, which were divided into three groups, and periodontitis. Initially, multivariate logistic regression models were used to assess the independent association between LE8 and periodontitis. In this study, Model I was not adjusted for covariates; Model II was only adjusted for sex, age, and race; and Model III was adjusted for annual family income, education level, marital status, alcohol consumption, floss use, depression, arthritis, asthma, cardiovascular disease, and gout on the basis of Model II. Next, the dose-response association between Life’s Essential 8 and periodontitis was assessed using smooth curve fitting. To determine the effect of confounders on the relationship between Life’s Essential 8 and periodontitis, subgroup analysis and interaction tests were performed. A *p*-value below 0.05 was considered indicative of statistical significance.

## Results

### Baseline characteristics of participants

A total of 9,039 participants with an average age of 52.16 ± 14.21 years were enrolled in this study, of whom 48.29% were male and 51.71% were female. The mean and standard deviation of LE8 was 66.29 ± 14.57. LE8 was categorized into three groups based on scores: low (LE8 < 50), moderate (50 ≤ LE8 < 80), and high (LE8 ≥ 80). The prevalence of periodontitis was 50.48% overall, and participants in the higher LE8 groups tended to have lower rates of periodontitis (Low 64.83%; Moderate 51.46%; High 31.38%; *p* < 0.001). Among the three groups of LE8 scores, statistically significant differences were observed in sex, age, race, annual family income, education level, marital status, alcohol consumption, floss use, depression, arthritis, asthma, cardiovascular disease, and gout (all *p* < 0.05) (Table 1).

**Table 1:** Baseline characteristics of participants (N = 9,039)

Life’s Essential 8 score (LE8)	Total	Low(LE8 < 50) N = 1217	Moderate(50 ≤ LE8 < 80) N = 6098	High(LE ≥ 80) N = 1724	*p*-value
**Age (year, mean ± SD)**	52.16 ± 14.21	55.03 ± 13.22	52.78 ± 14.29	47.95 ± 13.73	< 0.001
**Sex, n (%)**					< 0.001
Male	4365 (48.29%)	555 (45.60%)	3139 (51.48%)	671 (38.92%)	
Female	4674 (51.71%)	662 (54.40%)	2959 (48.52%)	1053 (61.08%)	
**Race, n (%)**					< 0.001
Mexican American	1264 (13.98%)	167 (13.72%)	903 (14.81%)	194 (11.25%)	
Other Hispanic	889 (9.84%)	122 (10.02%)	598 (9.81%)	169 (9.80%)	
Non-Hispanic White	4077 (45.10%)	486 (39.93%)	2724 (44.67%)	867 (50.29%)	
Non-Hispanic Black	1841 (20.37%)	386 (31.72%)	1283 (21.04%)	172 (9.98%)	
Others	968 (10.71%)	56 (4.60%)	590 (9.68%)	322 (18.68%)	
**Annual family income, n (%)**					< 0.001
Low	2356 (27.07%)	509 (43.21%)	1620 (27.58%)	227 (13.74%)	
Moderate	3530 (40.56%)	468 (39.73%)	2471 (42.07%)	591 (35.77%)	
High	2817 (32.37%)	201 (17.06%)	1782 (30.34%)	834 (50.48%)	
**Education, n (%)**					< 0.001
High school and below	3894 (43.13%)	741 (60.99%)	2748 (45.13%)	405 (23.49%)	
Above high school	5134 (56.87%)	474 (39.01%)	3341 (54.87%)	1319 (76.51%)	
**Marital status, n (%)**					< 0.001
Married	5337 (59.08%)	597 (49.10%)	3551 (58.27%)	1189 (69.01%)	
Widowed	638 (7.06%)	116 (9.54%)	464 (7.61%)	58 (3.37%)	
Divorced	1135 (12.57%)	185 (15.21%)	788 (12.93%)	162 (9.40%)	
Others	1923 (21.29%)	318 (26.15%)	1291 (21.18%)	314 (18.22%)	
**Alcohol consumption, n (%)**					< 0.001
Never	1127 (15.93%)	140 (16.13%)	745 (15.66%)	242 (16.68%)	
Moderate	3191 (45.10%)	331 (38.13%)	2098 (44.10%)	762 (52.52%)	
Severe	2758 (38.98%)	397 (45.74%)	1914 (40.24%)	447 (30.81%)	
**Floss use, n (%)**					< 0.001
Never	2760 (30.77%)	510 (42.32%)	1921 (31.77%)	329 (19.15%)	
Rarely	1472 (16.41%)	176 (14.61%)	991 (16.39%)	305 (17.75%)	
Moderately	1638 (18.26%)	172 (14.27%)	1097 (18.14%)	369 (21.48%)	
Frequently	3100 (34.56%)	347 (28.80%)	2038 (33.70%)	715 (41.62%)	
**Depression, n (%)**					< 0.001
Yes	855 (9.96%)	250 (21.57%)	534 (9.19%)	71 (4.39%)	
No	7730 (90.04%)	909 (78.43%)	5275 (90.81%)	1546 (95.61%)	
**Arthritis, n (%)**					< 0.001
Yes	2504 (27.76%)	556 (45.87%)	1697 (27.88%)	251 (14.58%)	
No	6517 (72.24%)	656 (54.13%)	4390 (72.12%)	1471 (85.42%)	
**Asthma, n (%)**					< 0.001
Yes	1243 (13.76%)	253 (20.82%)	808 (13.26%)	182 (10.56%)	
No	7788 (86.24%)	962 (79.18%)	5285 (86.74%)	1541 (89.44%)	
**Cardiovascular disease, n (%)**					< 0.001
Yes	760 (8.41%)	225 (18.49%)	476 (7.81%)	59 (3.42%)	
No	8279 (91.59%)	992 (81.51%)	5622 (92.19%)	1665 (96.58%)	
**Gout**					< 0.001
Yes	374 (4.14%)	96 (7.89%)	251 (4.12%)	27 (1.57%)	
No	8659 (95.86%)	1120 (92.11%)	5842 (95.88%)	1697 (98.43%)	
**Periodontitis, n (%)**					< 0.001
Yes	4563 (50.48%)	789 (64.83%)	3138 (51.46%)	549 (31.84%)	
No	4476 (49.52%)	428 (35.17%)	2960 (48.54%)	1175 (68.16%)	

### The association between LE8 and periodontitis

**Fig. 3:**
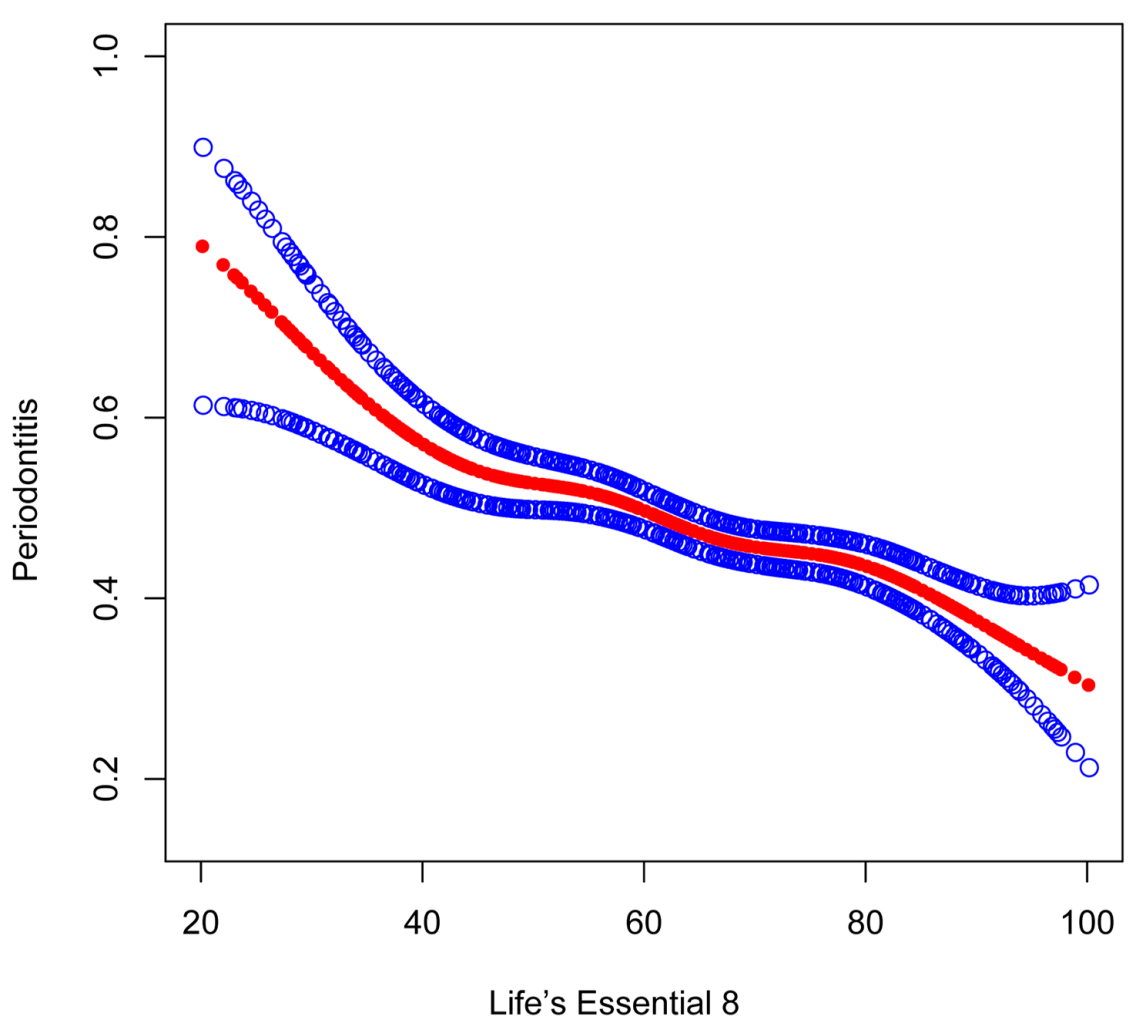
Dose-response relationship between LE8 and periodontitis

The findings revealed that the risk of periodontitis decreased with increasing LE8 score (Table 2). Both Model I (OR = 0.97;95% CI, 0.97–0.97, *p* < 0.001) and Model II (OR = 0.98;95% CI, 0.97–0.98, *p* < 0.001) showed that this connection was significant. In the model III, the relationship between the LE8 score and periodontitis remained consistent (OR = 0.98; 95% CI, 0.98–0.99, *p* < 0.001). After converting the LE8 score from a continuous variable to a categorical variable, a more significant result was observed. Compared with the lowest group, participants in the fully adjusted highest group had a significant reduction of 47% in the risk of developing periodontitis (OR = 0.53; 95% CI, 0.43–0.66, *P* < 0.001). Participants in the moderate group also showed a significantly lower risk of periodontitis than those in the lowest group (OR = 0.73;95% CI, 0.61–0.86, *p* < 0.001). Furthermore, smooth curve fitting, which exhibited a negative relationship in this study, was applied to assess the relationship between the LE8 score and periodontitis, as shown in Fig. 3.

**Table 2:** Association between LE8 and periodontitis in different models

Exposure	Model I (OR, 95% CI, *P*-value)	Model II (OR, 95% CI, *P*-value)	Model III (OR, 95% CI, *P*-value)
LE8 score	0.97 (0.97, 0.97) < 0.001	0.98 (0.97, 0.98) < 0.001	0.98 (0.98, 0.99) < 0.001
LE8 score, (groups)			
Low (LE8 < 50)	1.0	1.0	1.0
Moderate (50 ≤ LE8 < 80)	0.58 (0.51, 0.65) < 0.001	0.59 (0.51, 0.67) < 0.001	0.73 (0.61, 0.86) < 0.001
High (LE8 ≥ 80)	0.25 (0.22, 0.30) < 0.001	0.34 (0.29, 0.41) < 0.001	0.53 (0.43, 0.66) < 0.001

LE8, Life’s Essential 8; OR, odds ratio; CI, confidence interval

Model I was unadjusted

Model II was adjusted for sex; Age; and race

Model III adjusted for sex; age; race; annual family income; education level; marital status; floss use; alcohol consumption; depression; arthritis; asthma; cardiovascular disease; and gout

A threshold, nonlinear association between LE8 and periodontitis was found in a generalized additive model. The solid red line represents the smooth curve fit between variables. Blue bands represent the 95% confidence interval from the fit. All were adjusted for sex; age; race; annual family income; education level; marital status; floss use; alcohol consumption; depression; arthritis; asthma; cardiovascular disease; and gout.

### Subgroup analysis

The findings of the subgroup analysis also supported the negative correlation between the LE8 score and periodontitis (Table 3). We found significant interactions between the LE8 score and cardiovascular disease with the presence of periodontitis. The influence of LE8 on periodontitis was more significant in those who did not have cardiovascular disease (yes: OR 0.988, 95% CI 0.978–0.999, *P* = 0.028; no: OR 0.969, 95% CI 0.966–0.973, *P* < 0.001; *P* for interaction = 0.044). That is, with or without cardiovascular disease, the relationship between LE8 and periodontitis was different. Based on this result, we created Table 4 to further explore the relationship between LE8 and periodontitis stratified by cardiovascular disease. As shown in Table 4, in the fully adjusted model, the association between LE8 and periodontitis was nonsignificant in participants with cardiovascular disease (OR 0.998,95% CI 0.982–1.014, *p* = 0.802) and significant in those without cardiovascular disease (OR 0.984, 95% CI 0.980–0.989, *p* < 0.001). When the LE8 score was more than 80, the risk of periodontitis in participants without cardiovascular disease was 28.4% lower than that in participants with cardiovascular disease in Model III (with cardiovascular disease: OR 0.813, 95% CI 0.345–1.920, *p* = 0.637; without cardiovascular disease: OR 0.529, 95% CI 0.423-0,661, *p* < 0.001).

**Table 3:** Subgroup analysis for the association between LE8 and periodontitis

Subgroup	N	Periodontitis [OR (95% CI)]	*P* for interaction
Sex			0.198
Male	4365	0.969 (0.964, 0.973) < 0.001	
Female	4674	0.970 (0.966, 0.974) < 0.001	
Age			0.193
30–43	2967	0.970 (0.965, 0.976) < 0.001	
44–58	2959	0.969 (0.964, 0.974) < 0.001	
59–80	3113	0.982 (0.976, 0.987) < 0.001	
Education level			0.272
High school and below	3894	0.978 (0.973, 0.982) < 0.001	
Above high school	5134	0.973 (0.969, 0.977) < 0.001	
Annual family income			0.108
Low	2356	0.983 (0.977, 0.988) < 0.001	
Moderate	3530	0.977 (0.972, 0.982) < 0.001	
High	2817	0.964 (0.959, 0.970) < 0.001	
Marital status			0.706
Married	5337	0.966 (0.962, 0.970) < 0.001	
Widowed	638	0.987 (0.975, 0.999) 0.029	
Divorced	1135	0.978 (0.969, 0.986) < 0.001	
Others	1923	0.974 (0.968, 0.980) < 0.001	
Alcohol consumption			0.272
Never	1127	0.977 (0.969, 0.985) < 0.001	
Moderate	3191	0.970 (0.965, 0.975) < 0.001	
Severe	2758	0.966 (0.961, 0.972) < 0.001	
Floss use			0.311
Never	2760	0.972 (0.967, 0.978) < 0.001	
Rarely	1472	0.968 (0.960, 0.975) < 0.001	
Moderately	1638	0.969 (0.962, 0.976) < 0.001	
Frequently	3100	0.975 (0.970, 0.980) < 0.001	
Depression			0.929
Yes	855	0.976 (0.967, 0.985) < 0.001	
No	7730	0.968 (0.965, 0.971) < 0.001	
Arthritis			0.813
Yes	2504	0.979 (0.973, 0.984) < 0.001	
No	6517	0.967 (0.963, 0.970) < 0.001	
Asthma			0.418
Yes	1243	0.966 (0.959, 0.974) < 0.001	
No	7788	0.969 (0.966, 0.972) < 0.001	
Cardiovascular disease			0.044
Yes	760	0.988 (0.978, 0.999) 0.028	
No	8279	0.969 (0.966, 0.973) < 0.001	
Gout			0.261
Yes	374	0.982 (0.967, 0.998) 0.025	
No	8659	0.969 (0.966, 0.972) < 0.001	

**Table 4:** Association between LE8 and periodontitis stratified by cardiovascular disease

Exposure	Model I (OR, 95% CI, *P*-value)	Model II (OR, 95% CI, *P*-value)	Model III (OR, 95% CI, *P*-value)
**With cardiovascular disease**			
LE8 score	0.988 (0.978, 0.999) 0.028	0.986 (0.975, 0.997) 0.014	0.998 (0.982, 1.014) 0.802
LE8 score, (groups)			
Low (LE8 < 50)	1.0	1.0	1.0
Moderate (50 ≤ LE8 < 80)	0.893 (0.632, 1.263) 0.523	0.819 (0.569, 1.179) 0.283	0.851 (0.506, 1.432) 0.544
High (LE8 ≥ 80)	0.492 (0.273, 0.885) 0.018	0.437 (0.235, 0.813) 0.009	0.813 (0.345, 1.920) 0.637
**Without cardiovascular disease**			
LE8 score	0.969 (0.966, 0.973) < 0.001	0.976 (0.972, 0.979) < 0.001	0.984 (0.980, 0.989) < 0.001
LE8 score, (groups)			
Low (LE8 < 50)	1.0	1.0	1.0
Moderate (50 ≤ LE8 < 80)	0.575 (0.501, 0.662) < 0.001	0.578 (0.497, 0.670) < 0.001	0.727 (0.604, 0.874) 0.001
High (LE8 ≥ 80)	0.259 (0.219, 0.305) < 0.001	0.342 (0.286, 0.409) < 0.001	0.529 (0.423, 0.661) < 0.001

LE8, Life’s Essential 8; OR, odds ratio; CI, confidence interval

Model I was unadjusted

Model II was adjusted for sex; age; and race

Model III was adjusted for sex; age; race; annual family income; education level; marital status; floss use; alcohol consumption; depression; arthritis; asthma; and gout

## Discussion

A negative relationship between the LE8 score and the likelihood of periodontitis was observed in this cross-sectional investigation. The higher the LE8 score is, the lower the likelihood of periodontitis. Additionally, it is interesting to note that in stratified analyses, individuals with and without cardiovascular disease showed differences in the relationship between LE8 and periodontitis. Individuals with cardiovascular disease have a higher likelihood of developing periodontitis relative to those without cardiovascular disease. The underlying reasons may need to be explained by more research.

The application and value of Life’s Essential 8 has been universally confirmed as an indicator for assessing cardiovascular health. Sun J, and Li Y [37] reported that higher LE8 scores are associated with a reduced risk of all-cause and cardiovascular disease-specific mortality. Using data from the UK Biobank study, Petermann-Rocha F [38] observed that individuals with a lower LE8 score had a higher risk of MACEs (major adverse cardiovascular events) and individual cardiovascular outcomes. There is no doubt about the relationships between LE8 and cardiovascular disease. In addition, some scholars have found that LE8 is associated with chronic kidney disease and nonalcoholic fatty liver disease [19, 20]. However, studies on LE8 and periodontal disease have not been reported. Therefore, there is no reason not to explore the relationship between LE8 and periodontitis. If feasible, LE8 might also be an indicator for assessing oral health, especially periodontal health. We attempted to do this and spared no effort to explain how they might be related. The summarized possible causes are as follows.

On the one hand, LE8 contains four healthy behaviors (diet, physical activity, nicotine exposure, sleep health). The Healthy Eating Index (HEI-2015) was used to compute the diet score. Periodontitis is more likely to occur in people who have a lower total healthy eating index, according to research by Li XY [39]. Additionally, Iwasaki M, Usui M, and their associates have shown that insufficient sleep is associated with severe periodontitis in Japanese adults [40]. Han DH, and Kim MS’s findings suggested that extra long sleep, those who sleep 9 h or more, is a risk factor for periodontitis [41]. These findings are consistent with the criteria for sleep scoring in LE8. Additionally, in Almohamad M’s study, participants who engaged in more overall physical activity and less overall sedentary behavior had a lower prevalence of periodontal disease [42]. Moreover, smoking is the major preventable risk factor for periodontitis and has been generally confirmed [43–45].

On the other hand, the components of LE8 include 4 health factors (body mass index, blood lipids, blood glucose, and blood pressure). Diabetes mellitus (type 1 and type 2) is a known risk factor for periodontitis. According to results from mechanistic research, diabetes mellitus accelerates periodontal deterioration by causing a hyperinflammatory reaction to periodontal bacteria and impairing the healing process [46]. Metabolic syndrome, defined as a cluster of obesity, abnormal blood lipids, abnormal blood pressure and abnormal blood glucose, has been reported to be associated with periodontitis [47].

Overall, the elements contained in the LE8 score are inextricably linked to the risk of periodontitis. Therefore, it makes sense to use the LE8 score to assess periodontal health. It also serves as a reminder to maintain a healthy lifestyle, especially for those who are more susceptible to periodontal disease.

The data for this study came from the NHANES database, which has the advantage of being representative, publicly available, free, and easily accessible. However, it also has some limitations. First, causality cannot be determined by cross-sectional studies. Second, the NHANES database only contains data from Americans and is not typical of the demographics of other nations. Third, we cannot draw direct conclusions about the results of the study, and more prospective longitudinal studies are needed to confirm the study’s findings [48].

## Conclusion

This cross-sectional study revealed a negative relationship between the LE8 score and the risk of periodontitis. The results showed that LE8 would be useful as a practical and effective method of promoting periodontal health. It is necessary to conduct more studies on the causal and longitudinal relationships between LE8 and the risk of periodontitis.

### Electronic supplementary material

Below is the link to the electronic supplementary material.

Supplementary Material 1

## Data Availability

All the data can be found in the NHANES database (https://www.cdc.gov/nchs/nhanes/index/htm).

## Abbreviations

LE8: Life’s Essential 8
LE7: Life’s Essential 7
NHANES: National Health and Nutrition Examination Survey
CDC-AAP: The Centers for Disease Control and Prevention and the American Academy of Periodontology
CVH: Cardiovascular Health
CKD: Chronic Kidney Disease
NAFLD: Nonalcoholic Fatty Liver Disease
MEC: Mobile Examination Center
BMI: Body Mass Index
HEI: Healthy Eating Index
AL: Attached Level
PD: Probing Depth
NIAAA: National Institute on Alcohol Abuse and Alcoholism
OR: Odds Ratio
CI: Confidence Interval
PHQ-9: Patient Health Questionnaire-9
MACEs: Major Adverse Cardiovascular Events
